# Bilateral Acute Posterior Multifocal Placoid Pigment Epitheliopathy With Bacillary Layer Detachment Following Sinopharm COVID-19 Vaccination: A Case Report

**DOI:** 10.7759/cureus.55369

**Published:** 2024-03-02

**Authors:** P Salim Mahar, Mohammad Daniyal Monis, Abdul Sami Memon, Muhammad Azam

**Affiliations:** 1 Ophthalmology, Aga Khan University Hospital, Karachi, PAK; 2 Ophthalmology, Isra Postgraduate Institute of Ophthalmology, Karachi, PAK

**Keywords:** covid 19, coronavirus disease 2019, aluminum hydroxide, bacillary layer detachment, multiple placoid epitheliopathy, sinopharm vaccine

## Abstract

Various ocular manifestations associated with COVID-19 and vaccines, affecting both the anterior and posterior segments of the eye have been documented in the literature. In this report, we present the case of a 25-year-old male who complained of sudden-onset blurred vision and metamorphopsia in both eyes one day after receiving the second dose of the Sinopharm COVID-19 vaccine. The visual loss was painless, with no reported flashes or floaters. The patient had no significant medical or surgical history, no history of trauma, and no drug intake. Upon ocular examination, the best-corrected visual acuity was 6/60 (Snellen chart) in both eyes. The anterior segments appeared unremarkable, while fundoscopy revealed multiple yellowish-white subretinal lesions at the posterior pole of both eyes. Spectral domain optical coherence tomography (SD-OCT) confirmed the presence of subretinal fluid (SRF) with neurosensory detachment in each eye, along with bacillary layer detachment (BALAD). There were no signs of inflammation in the vitreous cavity. A diagnosis of acute posterior multifocal plaque pigment epitheliopathy (APMPPE) was established. The patient was prescribed nepafenac 0.1% drops to be instilled three times a day in both eyes and was advised to return for a follow-up examination in two weeks. At the follow-up visit, the patient's vision had improved to 6/9 in the right eye and 6/6 in the left eye, with most of the SRF absorbed. Unilateral APMPPE with BALAD has been mentioned in the literature following various COVID-19 vaccinations, but, to the best of our knowledge, this is the first case report where bilateral APMPPE with BALAD is reported. This case emphasizes the importance of a thorough eye examination for individuals experiencing ocular symptoms after receiving the COVID-19 vaccine.

## Introduction

The COVID-19 pandemic emerged as a sustained global health concern, stemming from the SARS-CoV-2 virus. According to the World Health Organization (WHO), there were 760,793,067 confirmed cases of the disease until March 2023 worldwide. In Pakistan, the cumulative number of COVID-19 cases was 1,579,006, resulting in the unfortunate loss of 6,904,443 lives globally and 30,648 in Pakistan [1].

In response to the pandemic, the WHO Strategic Advisory Group recommended the utilization of various vaccines against COVID-19. In Pakistan, the government predominantly provided the Sinopharm vaccine, an inactivated vaccine designed to stimulate the body's immune system without the risk of causing the disease. This vaccine is adjuvanted with aluminum hydroxide to enhance the immune system's response.

A study conducted by Meo and colleagues investigated the adverse effects of both the first and second doses of the Sinopharm vaccine on medical students and healthcare workers. The findings revealed that 61.3% of respondents experienced pain at the injection site, 40.6% reported general lethargy, 23.9% encountered myalgia/body pain, 22.4% had a low-grade fever, and 21% suffered from headaches [2].

Ng et al. commented on ocular adverse effects post-COVID-19 vaccination, encompassing facial and abducens nerve palsies, macular neuroretinopathy, central serous chorioretinopathy (CSCR), central retinal vein occlusion (CRVO), uveitis, and Grave’s disease [3].

Pang reported on 12 eyes of nine patients with ocular complaints, including choroiditis, uveitis, keratitis, scleritis, acute retinal necrosis, and iridocyclitis. Notably, all patients developed ocular symptoms within 14 days after receiving the inactivated COVID-19 vaccine [4].

The interaction between the effects of the disease and vaccination often exhibits overlap. Various adjuvants are employed in vaccinations to bolster the patient's immune response and secure adequate immunity. Despite these observations, the exact mechanism underlying ocular side effects following COVID-19 vaccines remains elusive. Different mechanisms have been postulated, ranging from direct infection by attenuated viruses to inflammation induced by adjuvant compounds [5].

In summary, the dynamic landscape of COVID-19 vaccination presents both challenges and opportunities. Vigilant monitoring and research efforts are crucial to understanding the multifaceted effects and ensuring the safety and efficacy of vaccination programs globally.

## Case presentation

A 25-year-old male presented at the eye clinic with a complaint of decreased distance and near vision in both eyes over the previous two weeks. The onset was gradual, painless, and accompanied by distortion of images. No history of trauma or drug use was reported, and the patient's general health was unremarkable. Notably, he had received the second dose of the Sinopharm COVID-19 vaccine one day before the onset of ocular symptoms.

**Figure 1:**
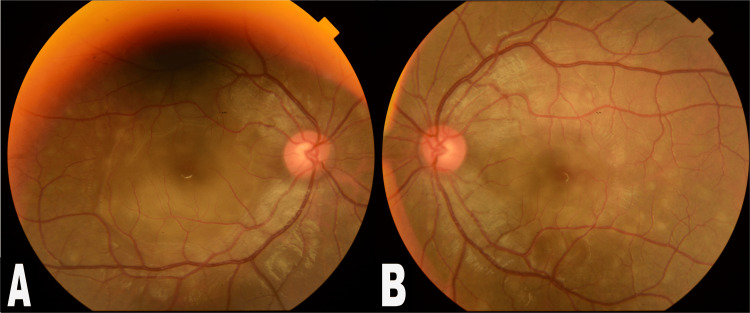
Fundus photographs of the posterior poles of both eyes: (A) right eye and (B) left eye The photographs of the fundus show multiple yellowish-round lesions at the level of the retinal pigment epithelium with elevation of the neurosensory retina in both eyes.

Upon examination, his best-corrected visual acuity was measured at 6/60 (Snellen chart) in both eyes (right: +1.75/-1.00DC@020, left: +1.50/-1.25DC@015). Pupillary reactions were brisk, and full eye movements were observed. Anterior segment biomicroscopy revealed no abnormalities, with intraocular pressure (IOP) measured at 14 mmHg in both eyes using the Goldmann applanation tonometer. A dilated fundus examination unveiled multiple yellowish-white round lesions at the retinal pigment epithelium level over the posterior pole of both eyes. Correspondingly, there was an elevation of the retina with the presence of subretinal fluid (SRF) (Figure 1).

**Figure 2:**
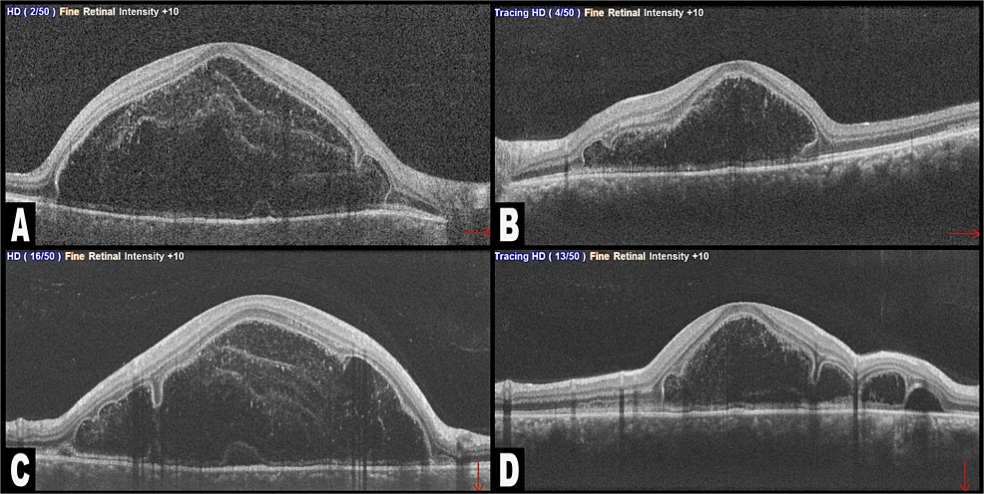
Spectral domain optical coherence tomography (SD-OCT) of both eyes: (A) horizontal section of the right eye; (B) horizontal section of the left eye; (C) vertical section of the right eye; (D) vertical section of the left eye The SD-OCT images show bacillary layer detachment (BALAD) in each eye, seen as a clear separation between the hyporeflective photoreceptor myoid zone (MZ) and ellipsoid zone (EZ), along with marginal septa and serous retinal detachment. The anterior border/ceiling of the BALAD in both eyes manifests as a granular hyperreflective band, while the posterior border/floor seamlessly continues with variable thickness and reflectivity within the adjacent retina’s EZ. Notably, a V-shaped granular hyperreflective band forms at the anterior border/ceiling of the BALAD in the left eye.

Optic discs and retinal vessels appeared within normal limits, and no cellular activity was noted in the vitreous cavity. Spectral domain optical coherence tomography (SD-OCT) of the posterior pole confirmed these findings, along with bacillary layer detachment (BALAD) (Figure 2).

**Figure 3:**
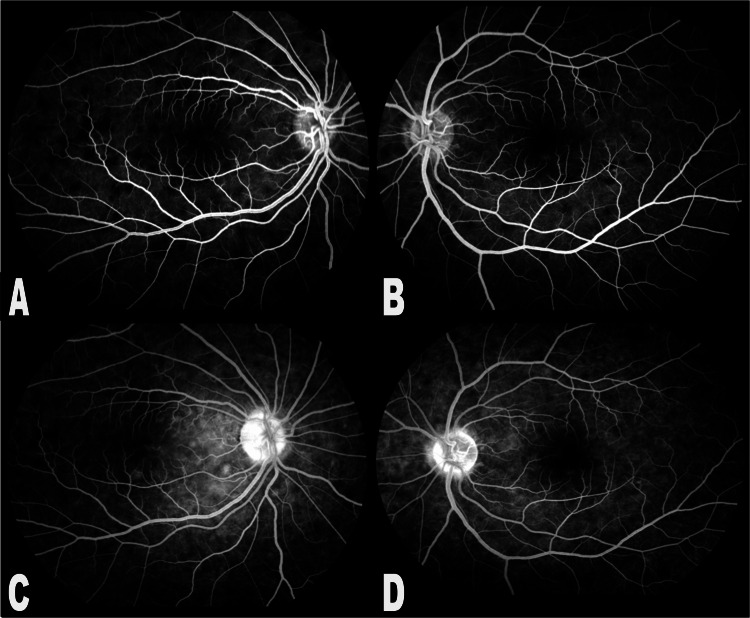
Fluorescein angiography of both eyes: (A and B) early phase shows masking of dye; (C and D) late phase shows hyperfluorescence

Fundus fluorescein angiography (FFA) of both eyes revealed early fluorescence due to the masking effect, with hyperfluorescence in the late phase due to fluorescein staining (Figure 3).

A clinical diagnosis of acute posterior multifocal placoid pigment epitheliopathy (APMPPE) was entertained along with BALAD. Baseline hematological investigations, including complete blood count, liver function tests, serum cortisol level, C-reactive protein, blood urea, and serum creatinine, were all within normal limits.

**Figure 4:**
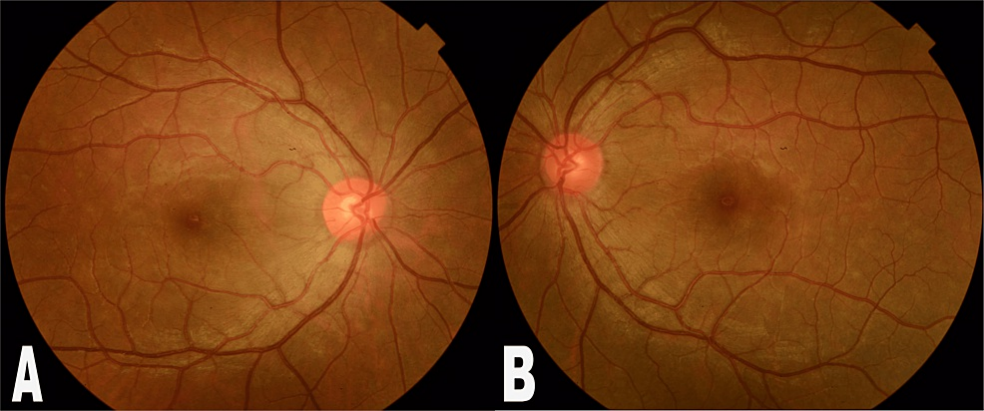
Follow-up fundus photographs of the posterior pole of both eyes: (A) right eye; (B) left eye The photographs of the fundus show resolved pigmentary changes at follow-up.

**Figure 5:**
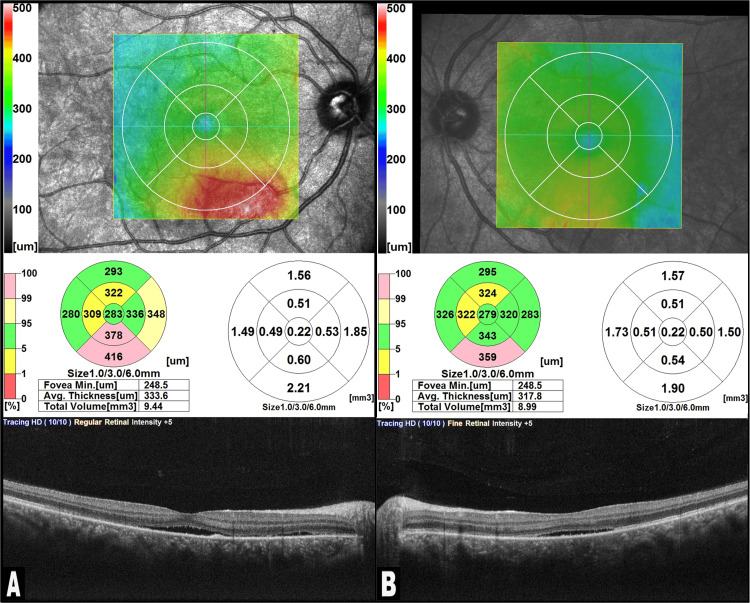
Follow-up spectral domain optical coherence tomography (SD-OCT) of both eyes: (A) right eye; (B) left eye The SD-OCT of the right and left eye after two weeks shows a decrease in subretinal fluid with reattachment of the bacillary layer detachment (BALAD) and the return of almost normal anatomy.

The patient was prescribed nepafenac 0.1% drops (Nevanac, Novartis Pharmaceuticals, Karachi, Pakistan) to be instilled three times a day in both eyes and advised to return to the eye clinic in two weeks. At the follow-up examination, his visual acuity had improved to 6/9 in the right eye and 6/6 in the left eye on the Snellen chart (right eye: +0.25/-0.50DC@105, left eye: +0.75/-0.25DC@160). Anterior segments and IOP were within the normal range. Fundus examination indicated a significant decrease in SRF at the posterior pole (Figure 4) which was confirmed by SD-OCT examination (Figure 5).

## Discussion

The presented case report underscores an intriguing occurrence of ocular manifestations following Sinopharm COVID-19 vaccination, contributing to the expanding literature on vaccine-related complications. The patient, a 25-year-old male, exhibited sudden, painless visual impairment and metamorphopsia one day after the second vaccine dose. The clinical examination revealed a best-corrected visual acuity of 6/60 in both eyes, with distinctive yellowish-white subretinal lesions at the posterior pole. The SD-OCT images confirmed SRF with neurosensory detachment with BALAD, leading to the diagnosis of bilateral APMPPE with BALAD.

This case adds to the growing body of evidence regarding ocular manifestations associated with COVID-19 vaccines. The discussion encompasses several key aspects, including the global context of the COVID-19 pandemic, the utilization of the Sinopharm vaccine in Pakistan, reported adverse effects, a review of ocular manifestations post vaccination, and potential mechanisms underlying these ocular side effects.

The COVID-19 pandemic prompted global efforts to develop vaccines, with Sinopharm emerging as a common choice in Pakistan. Meo and colleagues investigated Sinopharm's adverse effects, reporting common side effects aligning with general responses to various COVID-19 vaccines [2,6].

Ocular manifestations post-COVID-19 vaccination have been documented, spanning conditions like facial and abducens nerve palsy, macular neuroretinopathy, CSCR, CRVO, uveitis, and Grave’s disease. The presented case aligns with these reports, specifically describing APMPPE, an inflammatory eye condition affecting the posterior pole [3-5,7,8].

Pang et al. reported diverse ocular complaints within 14 days after receiving inactivated COVID-19 vaccines, highlighting the complexity of the relationship between vaccination and ocular manifestations [4].

The discussion acknowledges the challenge of distinguishing between disease-related and vaccine-induced effects. Vaccines utilize different adjuvants, and their interactions with the host may contribute to adverse events as well. Despite these observations, the precise mechanisms underlying ocular side effects following the COVID-19 vaccination remain unknown.

Moving to the unique aspect of this case, it details an isolated hyperacute bilateral presentation of APMPPE with BALAD, a phenomenon not previously reported.

In 1968, Gass first described APMPPE as an inflammatory chorioretinopathy, part of the white-dot syndrome. Though uncommon with no gender predilection, it typically occurs bilaterally and is self-limiting. It is characterized by an acute bilateral decrease of central vision with creamy-yellow or grey-white placoid lesions at the retinal pigment epithelium layer (RPE) level in the posterior pole. It mostly affects individuals between the second and fourth decades of life, often associated with a history of flu-like or viral illness in one-third of the cases. Various vaccinations, including COVID-19 vaccines, have been known to cause the disease irrespective of their active agent or mode of action [9,10].

While attempts have been made to explain the etiology, the most recent hypothesis by Steptoe et al. proposes a direct neurotrophic infection. It suggests that retinal nerve fiber layer (RNFL) changes, along with the axonal spheroid formation of Henle's fiber layer, precede the outer retinal layer changes [11].

The pathogenesis remains not fully understood, but inflammation of the RPE and the outer retina as a cause of the characteristic lesions was earlier proposed by Gass [10]. Van Bushrik et al. suggested occlusive vasculitis of the choriocapillaris as the cause of the ischemic insult to the outer retina, leading to the appearance of the lesions. Subsequently, choroidal and outer retinal atrophy follow [12].

Recent reports indicate APMPPE occurring with BALAD, presumably, due to underlying ischemia causing photoreceptor layer stress and splitting. This phenomenon has been observed following both the COVID-19 infection and the COVID-19 vaccination [13-15].

Polyak, in 1941, named the basilar layer representing the inner segment- outer segment junction (IS-OS) of the photoreceptors seen as the ellipsoid zone on SD-OCT [16]. Its detachment, known as basilar layer detachment, is observed on SD-OCT as splitting between the ellipsoid zone with hyperreflective septa in between [17].

Clinically, basilar layer detachment appears as a dome-shaped serous retinal detachment, similar to CSCR. However, careful examination of SD-OCT reveals separation in-between the ellipsoid zone while the photoreceptor outer segments remain attached to the RPE, appearing as deposits on the RPE. The underlying choroid and posterior hyaloid face may appear normal. Lesions exhibit early hypofluorescence on FFA, followed by late marginal hyper- or hypofluorescence, leakage, and staining. On indocyanine angiography, the lesions appear to have hypocyanescence with or without juxtapapillary hypocyanescence [14,18].

Visual recovery has been linked to the flattening and reattachment of the split basilar layer, occurring in the sequence of the external limiting membrane, ellipsoid zone, and outer segment retinal pigment epithelium junction attachment [13]. Although both APMPPE and BALAD typically resolve spontaneously and exhibit favorable recovery, there is currently no unanimous agreement on their treatment approaches.

## Conclusions

This case contributes valuable insights to the evolving understanding of ocular manifestations associated with COVID-19 vaccination, emphasizing the importance of comprehensive eye examinations post vaccination. Further research is needed to elucidate mechanisms, guide healthcare professionals, and enhance patient care in the context of mass vaccination campaigns.
